# Supplementary material for: ONIX: a unified open-source platform for multimodal neural recording and perturbation during naturalistic behavior
Source: Nat Methods. 2024 Nov 11;22(1):187–92. doi: 10.1038/s41592-024-02521-1 (PMC11725498; doi:10.1038/s41592-024-02521-1)
Supplement: Supplementary file 1 — Supplementary Table 1. [file 41592_2024_2521_MOESM1_ESM.pdf]

nature methods

Supplementary information https://doi.org/10.1038/s41592-024-02521-1

ONIX: a unified open-source platform for multimodal neural recording and perturbation during naturalistic behavior

In the format provided by the authors and unedited

Supplementary Table 1

JP Newman, J Zhang, A Cuevas-López, NJ Miller, T Honda, MSH van der Goes, AH Leighton, F Carvalho, G Lopes, A Lakunina, JH Siegle, MT Harnet, MA Wilson, J Voigts: ONIX: A unified open-source platform for multimodal neural recording and perturbation during naturalistic behavior

	**Intan / 'Classic' Open Ephys**	**Neuropixels (Ver. 1.0)**[43]	**UCLA Miniscope**[45]	**ONIX**
**Headstage**				
Connection to host	SPI via LVDS twisted pairs	Coax or twisted pair	Coax or twisted pair	Coax
Onboard processing	None	None	None	FPGA
Connector scaling	8 additional conductors per chip	Limited to one probe, (two with ver. 2.0)	Limited to single camera sensor	Depends on SERDES choice, currently 150MB/s per coax (Ext. Fig. 7)
Arbitrary chip support	No	No	No	Yes
**Host Interface**				
Connection to PC	USB	PXI, PCIe	USB	PCIe
Communication protocol	Opal-Kelly Front-Panel + Drivers	Ad-hoc serialization	USB-webcam	Probe-agnostic serialization
Programming interface	Rhythm API (Intan chips only)	NeuroPixels-specific API	Cypress SDK for webcams	Probe-agnostic ONI API
Firmware	Rhythm Verilog (Intan chips only)	Neuropixels-specific	Cypress Firmware	ONI IP block + arbitrary probe-specific firmware
**Software Support**	Open Ephys GUI, Bonsai, Intan RHX	Open Ephys GUI, SpikeGLX	Miniscope GUI, Bonsai	Open Ephys GUI, Bonsai, ONIX console app
**Closed Loop Latency**	10s of milliseconds	~3 milliseconds	10s of milliseconds	<100 microseconds
**Compatibility Limitation**	Only supports Intan Chips	Only supports Neuropixels	Only supports camera sensors	Compatible with Intan chips, Camera sensors, Neuropixels, and any other sensors

Supplementary Table 1: Summary of communication data-acquisition architectures, host interfaces, software-support, closed-loop performance, and notable limitations for three widely used open source hardware projects for in-vivo electrophysiology.
