# Supplementary material for: ONIX: a unified open-source platform for multimodal neural recording and perturbation during naturalistic behavior
Source: Nat Methods. 2024 Nov 11;22(1):187–92. doi: 10.1038/s41592-024-02521-1 (PMC11725498; doi:10.1038/s41592-024-02521-1)
Supplement: Supplementary file 2 — Reporting Summary [file 41592_2024_2521_MOESM2_ESM.pdf]

nature portfolio

Corresponding author(s): Jakob Voigts

Last updated by author(s): Aug 5, 2024

Reporting Summary

Nature Portfolio wishes to improve the reproducibility of the work that we publish. This form provides structure for consistency and transparency in reporting. For further information on Nature Portfolio policies, see our Editorial Policies and the Editorial Policy Checklist.

Statistics

For all statistical analyses, confirm that the following items are present in the figure legend, table legend, main text, or Methods section.

n/a	Confirmed	
☐	☒	The exact sample size (*n*) for each experimental group/condition, given as a discrete number and unit of measurement
☐	☒	A statement on whether measurements were taken from distinct samples or whether the same sample was measured repeatedly
☐	☒	The statistical test(s) used AND whether they are one- or two-sided<br>*Only common tests should be described solely by name; describe more complex techniques in the Methods section.*
☐	☒	A description of all covariates tested
☒	☐	A description of any assumptions or corrections, such as tests of normality and adjustment for multiple comparisons
☐	☒	A full description of the statistical parameters including central tendency (e.g. means) or other basic estimates (e.g. regression coefficient) AND variation (e.g. standard deviation) or associated estimates of uncertainty (e.g. confidence intervals)
☐	☒	For null hypothesis testing, the test statistic (e.g. *F*, *t*, *r*) with confidence intervals, effect sizes, degrees of freedom and *P* value noted<br>*Give P values as exact values whenever suitable.*
☒	☐	For Bayesian analysis, information on the choice of priors and Markov chain Monte Carlo settings
☒	☐	For hierarchical and complex designs, identification of the appropriate level for tests and full reporting of outcomes
☒	☐	Estimates of effect sizes (e.g. Cohen's *d*, Pearson's *r*), indicating how they were calculated

Our web collection on statistics for biologists contains articles on many of the points above.

Software and code

Policy information about availability of computer code

Data collection	Data was collected using our own software (github repositories are referred to in the text), and software that we wrote as part of the Bonsai programming language (also available on github and referenced in the text), as described in the manuscript. All source code is available as stated in the text.
Data analysis	Data plotting was performed in Matlab, versions 2023 and 2024, and Python (ver. 3.6.15) no custom algorithms were used.

For manuscripts utilizing custom algorithms or software that are central to the research but not yet described in published literature, software must be made available to editors and reviewers. We strongly encourage code deposition in a community repository (e.g. GitHub). See the Nature Portfolio guidelines for submitting code & software for further information.

Data

Policy information about availability of data

All manuscripts must include a data availability statement. This statement should provide the following information, where applicable:

- Accession codes, unique identifiers, or web links for publicly available datasets
- A description of any restrictions on data availability
- For clinical datasets or third party data, please ensure that the statement adheres to our policy

Experimental data from Figure 3 is available on figshare at 10.6084/m9.figshare.26391160. Additional example data recorded with the system can be made available upon request.

Human research participants

Policy information about studies involving human research participants and Sex and Gender in Research.

Reporting on sex and gender	N/A
Population characteristics	N/A
Recruitment	N/A
Ethics oversight	N/A

Note that full information on the approval of the study protocol must also be provided in the manuscript.

Field-specific reporting

Please select the one below that is the best fit for your research. If you are not sure, read the appropriate sections before making your selection.

☒ Life sciences ☐ Behavioural & social sciences ☐ Ecological, evolutionary & environmental sciences

For a reference copy of the document with all sections, see nature.com/documents/nr-reporting-summary-flat.pdf

Life sciences study design

All studies must disclose on these points even when the disclosure is negative.

Sample size	We applied sample sized commonly used to assess whether a data acquisition system functions reliably. No power size calculations were used for this.
Data exclusions	No data exclusion was applied.
Replication	This study is based on performance metrics achievable with a new method. Other than the sample sizes used to ensure the robustness of these metrics, replication is no applicable in this context.
Randomization	Randomization is not applicable to a study establishing the basic function of a new method.
Blinding	There were no significant interactions between the users and the research animals that could be blinded.

Reporting for specific materials, systems and methods

We require information from authors about some types of materials, experimental systems and methods used in many studies. Here, indicate whether each material, system or method listed is relevant to your study. If you are not sure if a list item applies to your research, read the appropriate section before selecting a response.

Materials & experimental systems

n/a	Involved in the study	
☒	☐	Antibodies
☒	☐	Eukaryotic cell lines
☒	☐	Palaeontology and archaeology
☐	☒	Animals and other organisms
☒	☐	Clinical data
☒	☐	Dual use research of concern

Methods

n/a	Involved in the study	
☒	☐	ChIP-seq
☒	☐	Flow cytometry
☒	☐	MRI-based neuroimaging

Animals and other research organisms

Policy information about studies involving animals; ARRIVE guidelines recommended for reporting animal research, and Sex and Gender in Research

Laboratory animals	ChAT-IRES-Cre transgenic, PWK/PhJ x C57bl6, and C57bl6, all Jackson labs. Mice were aged between 8 and 14 months, and both male and female mice were used. Animals were housed in accordance with institutional guidelines approved by the internal IACUC at the

	Allen institute, MIT and HHMI Janelia research campus, in compliance with the standards set forth by the Association for Assessment and Accreditation of Laboratory Animal Care. This includes maintaining temperatures of 65-75°F (~18-23°C) with 40-60% humidity.
Wild animals	No wild animals were used in this study.
Reporting on sex	No effects related to sex were considered in this study.
Field-collected samples	No field collected samples were used in this study.
Ethics oversight	Massachusetts Institute of Technology (MIT), Allen Institute, and Janelia Institutional Animal Care and Use Committees.

Note that full information on the approval of the study protocol must also be provided in the manuscript.
